# The epidemiological trends of biliary tract cancers in the United States of America

**DOI:** 10.1186/s12876-022-02637-8

**Published:** 2022-12-29

**Authors:** Yong Jiang, Liyong Jiang, Feiyu Li, Qingbin Li, Shuai Yuan, Songhan Huang, Yingda Fu, Xiangyu Yan, Ji Chen, Hongxin Li, Shenhao Li, Jun liu

**Affiliations:** 1 Department of Liver Transplantation and Hepatobiliary Surgery, Shandong Provincial Hospital, Shandong University, No.324, Jingwu Road, Jinan, Shandong China; 2 Department of Liver Transplantation and Hepatobiliary Surgery, Shandong Provincial Hospital Affiliated to Shandong First Medical University, Jinan, China

**Keywords:** Biliary tract cancers (BTCs), Intrahepatic cholangiocarcinoma (ICC), Extrahepatic cholangiocarcinoma (ECC), Gallbladder cancer (GBC), Ampulla of Vater cancer (AVC)

## Abstract

**Background:**

Biliary tract cancers (BTCs) are a series of heterogeneous malignancies that are broadly grouped based on the anatomical site where they arise into subtypes including intrahepatic cholangiocarcinoma (ICC), extrahepatic cholangiocarcinoma (ECC), gallbladder cancer (GBC), and ampulla of Vater cancer (AVC).

**Methods and Results:**

The present study provides an overview of the epidemiology of the various BTCs based on data from the National Cancer Institute’s Surveillance, Epidemiology, and End Results (SEER) database from 2000 to 2018. Distinct differences in both incidence and mortality rates were observed for these BTCs as a function of age, sex, ethnicity, and calendar year. In 2018, BTCs emerged as the fifth most prevalent form of alimentary tract cancer in the USA. While the incidence and mortality of ICC appear to be increasing, the incidence rates of GBC, ECC, and AVC have remained stable, as have the corresponding mortality rates. The most common and deadliest BTCs in 2018 were ICC and GBC among males and females, respectively. The ethnic groups exhibiting the highest incidence rates of these different BTCs were American Indians and Alaska Natives for GBC, and Asian and Pacific Islanders for ICC, ECC, and AVC. The incidence of all of these forms of BTC rose with age. There were some variations in BTCs in terms of staging, locoregional surgical treatments, adjuvant therapies, and prognostic outcomes from 2000 to 2018.

**Conclusions:**

The epidemiological characteristics, staging, locoregional surgical treatments, adjuvant therapies, and prognostic outcomes were distinct for each of these BTCs.

**Supplementary Information:**

The online version contains supplementary material available at 10.1186/s12876-022-02637-8.

## Introduction

Biliary tract cancers (BTCs) are a series of heterogeneous malignancies that are broadly grouped based on the anatomical site where they arise into subtypes including intrahepatic cholangiocarcinoma (ICC), extrahepatic cholangiocarcinoma (ECC), gallbladder cancer (GBC), and ampulla of Vater cancer (AVC) [1, 2]. These different forms of BTC exhibit distinct epidemiological, etiological, and molecular characteristics that contribute to differences in their clinical presentation and treatment [3]. Overall, BTCs account for ~ 3% of all gastrointestinal neoplasms and are the sixth most common cancer of the alimentary tract after colorectal, pancreatic, liver, stomach, and esophageal cancers [4, 5]. Although rare, the prognosis for patients affected by BTCs is poor, with a 5-year overall survival (OS) rate of just 10–40% even after surgical tumor resection [6–10].

Several recent studies have suggested that BTC incidence is rising throughout the globe, with some changes in the prevalence of these tumors and their associated mortality rates in recent decades [11, 12]. Strikingly, the incidence of ICC has risen substantially, with an average annual increase of 4.36% over the last 10 years [13, 14]. Many researchers have also demonstrated the association of survival with adjuvant therapy in BTC patients [10, 15–18]. However, at present there is a lack of publications providing an updated overview of the epidemiological characteristics of BTC and associated patient survival outcomes.

The present study was thus developed as a population-based analysis of data derived from the Surveillance, Epidemiology, and End Results (SEER) database produced by the National Cancer Institute (https://seer.cancer.gov/) Program. Here, the trends of epidemiology, clinical characteristics, and prognostic findings associated with BTCs in USA from 2000 to 2018 are surveyed in detail.

## Materials and methods

The SEER 18 registry serves as a definitive source for cancer-related statistics corresponding to ~ 28% of the population of the USA. These data were used to assess the BTC incidence and mortality rates for the period between 2000 and 2018 using the SEER*Stat software (v 8.4.0). Data were obtained for intrahepatic bile duct (C22.1), extrahepatic bile duct (C24.0), gallbladder (C23.9), ampulla of Vater (C24.1), and other biliary tract cancers (C24.8 and C24.9). Hematological cancers were excluded from this analysis. The overall incidence rates of alimentary tract cancers were also analyzed, as were the demographic, tumor characteristics, treatment, and survival details for all BTC cases diagnosed in the 2000–2018 period. While Klatskin tumors were misclassified as ICC in the ICD-O-2 and as ICC or ECC in ICD-O-3, for the present study liver and intrahepatic Klatskin tumors (histology code 8162/3) were treated as ECC cases given that these tumors are of extrahepatic origin [19]. As the BTC staging criteria published by the AJCC changed significantly from 2000 to 2018, the SEER staging system was instead used for the present analysis, and tumors were staged as cases of localized, regional, or distant disease. Cases were grouped into four categories in accordance with the surgical and adjuvant therapy approaches employed, including no surgery or adjuvant therapy, surgery without adjuvant therapy, adjuvant therapy without surgery, and both surgery and adjuvant therapy.

### Statistical analysis

Categorical data are given as counts with percentages. BTC incidence rates were compared to those of other forms of alimentary tract cancer. Incidence and incidence-based mortality rates for different BTC subtypes were reported as a function of sex, ethnicity, age, and calendar year, and incidence rate ratios in 2000–2018 are reported as a function of by sex or race. The annual percentage change (APC) in BTC incidence and incidence-based mortality was analyzed using Joinpoint (v 4.9.0.0) based on the piecewise log-linear time calendar trends [20]. Similarly, the average age-specific percentage change (AAPC) was calculated to assess the trend in age. The estimated 2-year relative survival rates were also compared among patients with different BTC subtypes and across calendar years. Multivariate analyses were conducted using a Cox proportional hazards regression model. SPSS 26.0 was used for all statistical testing, and a two-sided *P* < 0.05 was considered significant.

## Results

Between 2000 and 2018, 64,666 cases of BTC were identified. These included 14,830 cases of ICC (22.9%), 17,004 of ECC (26.3%), 19,187 of GBC (29.7%), 9,742 of AVC (15.1%), and 3,903 cases of other biliary tract cancers (6.0%) (Additional file 1: Table S1). In total, 661 Klatskin tumors (18 coded as in the liver, 643 as ICC) were identified and recategorized as ECC for the purposes of this analysis. Of these identified BTC cases, 53.6% (34,691) and 46.4% (29,975) were diagnosed in females and males, were diagnosed in females and males, respectively.

The mean patient age at time of diagnosis was 70.03 ± 12.96 years, with a median age of 71 years (range: 1–100 years). Of these patients, 98.4% were 40 years of age or older (63,641 cases). Overall, 78.2% (50,589) of patients were white, 9.5% [6] were black, 11.1% [7] were Asians and Pacific Islanders, 1% (625) were American Indians and Alaskan Natives, and 0.3% (171) were of unknown race. Of the 30,266 BTC cases for which a known grade was available (46.8%), 4125 (6.4%), 13,558 (21%), 11,909 (18.4%), and 674 (1.0%) were grade I, grade II, grade III, and grade IV, respectively. Of the 56,262 BTC cases for which staging information was available (87.0%), 11,299 (17.5%) were localized, 22,993(35.6%) were regional, and 21,970 (34%) were classified as distant disease when initially diagnosed. With respect to treatment, 25,514 cases (39.5%) did not undergo surgery or adjuvant therapy, while 15,158 (23.4%) underwent surgery alone, 1350 (20.9%) underwent adjuvant treatment alone, and 10,489 (16.2%) underwent both surgery and adjuvant treatment.

**Fig. 1:**
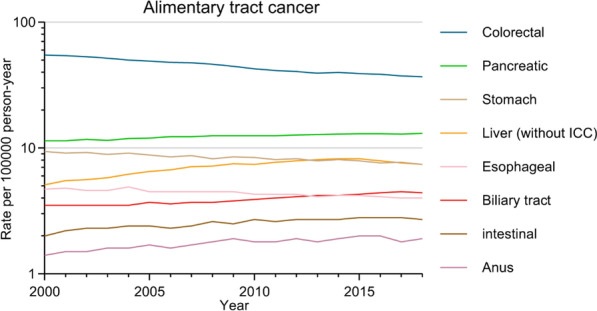
Incidences of alimentary tract cancers, 2000–2018

**Fig. 2:**
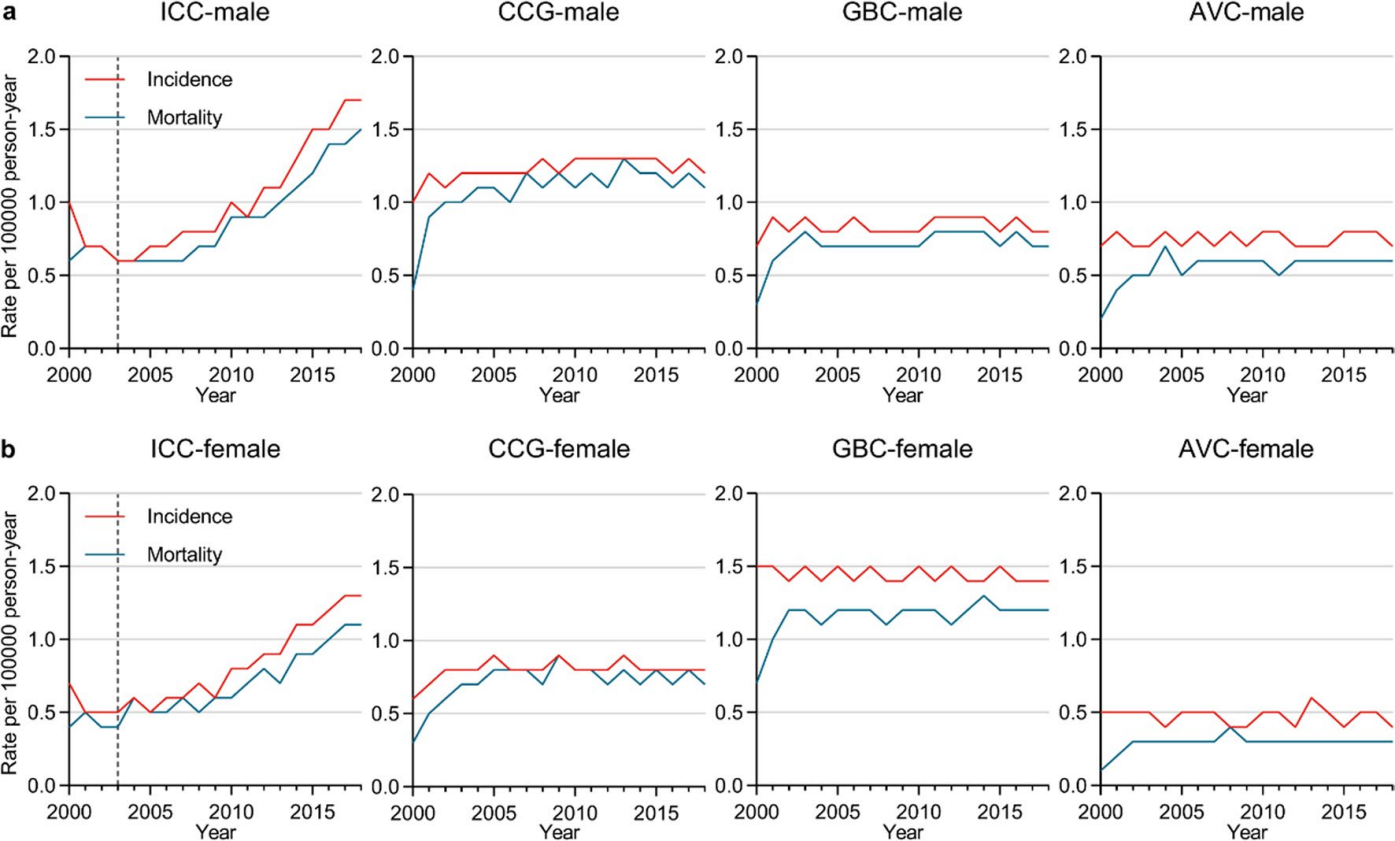
(**a**) Incidence and incidence-based mortality for BTCs among males, 2000–2018. (**b**) Incidence and incidence-based mortality for BTCs among females, 2000–2018

**Fig. 3:**
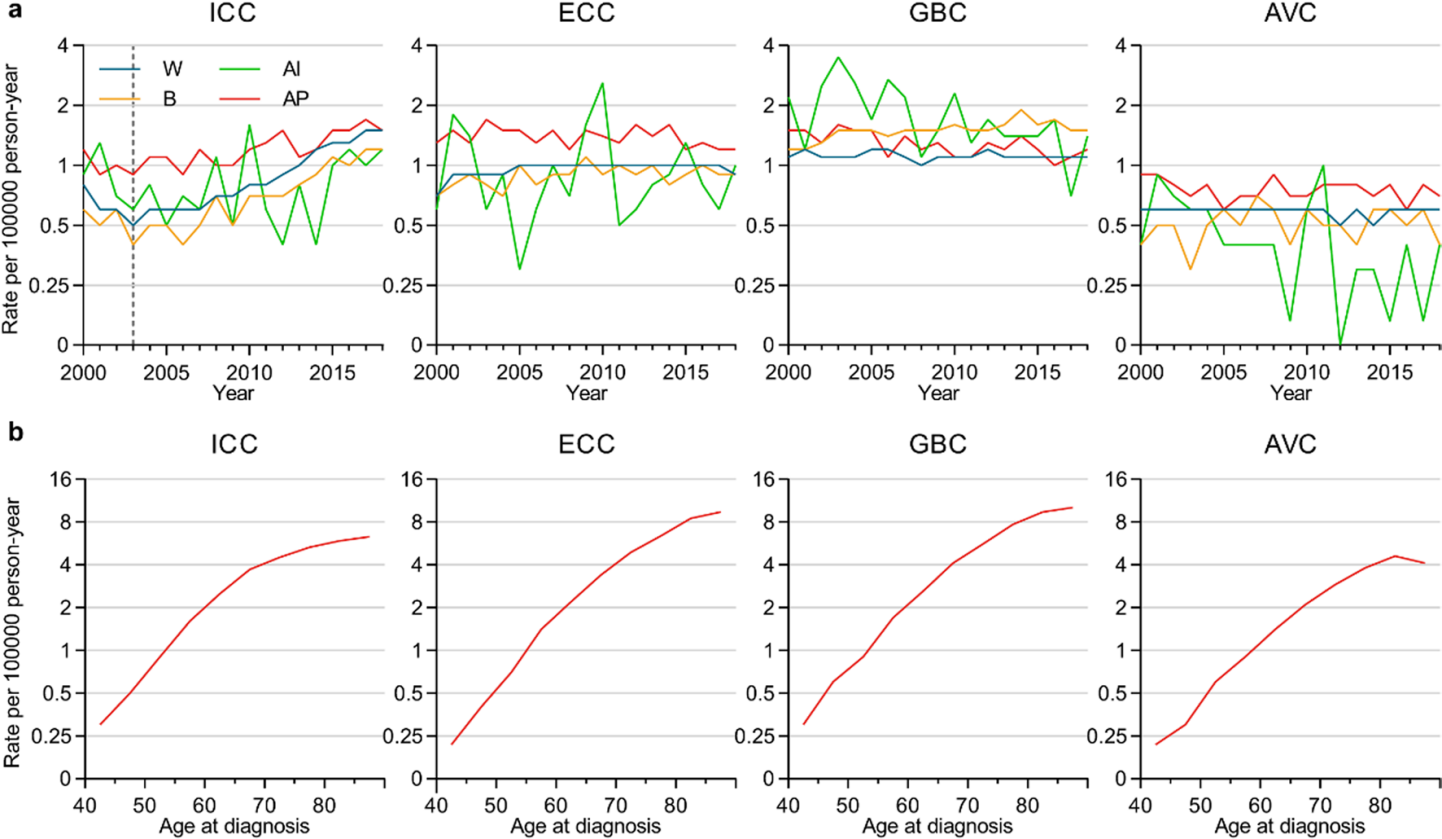
(**a**) Incidence of BTCs as a function of ethnicity, 2000–2018. (**b**) Incidence of BTCs as a function of age (≥ 40 years). *W* White, *B* Black, *AI* American Indian and Alaskan Native, *AP* Asian and Pacific Islanders

Next, age-adjusted incidence and incidence-based mortality rates (per 100,000 persons) were calculated for the different BTCs based on the population data derived from the SEER database with reference to the standard population of the USA in 2000. Overall, the BTC incidence rates were 3.5 and 4.4 per 100,000 persons in 2000 and 2018, respectively, with an APC of 1.4 (95% CI, 1.1–1.8) from 2004 to 2018, ranking as the sixth and fifth most common alimentary tract cancers in 2000 and 2018, respectively (Fig. 1). Incidence and incidence-based mortality rates, as well as incidence rate ratios for the different forms of BTC among males and females were next compared (Fig. 2a, b and Additional file 1: Table S2), revealing that incidence of GBC was higher among females (ratio of male: female incidence, 1: 1.556, between 2000 and 2018), whereas the incidence of ICC, ECC and AVC was higher among males (incidence rate ratio of male: female, 1: 0.727, 1: 0.667, and 1: 0.714 for ICC, ECC, and AVC, respectively. between 2000 and 2018). In 2018, the highest BTC incidence and mortality rates were for ICC in males and GBC in females. From 2003 to 2018, significant increases in both the incidence and incidence-based mortality rates were observed in patients with ICC, with APC incidences of 7.7 (95% CI, 6.9–8.5) and 7.0 (95% CI, 6.0–8.0) for males and females, respectively, and the corresponding incidence-based mortality rates for APC of 6.4 (95% CI, 4.8–8.1) and 6.3 (95% CI, 5.0–7.6) for males and females, respectively. Overall trends in BTC incidence as a function of ethnicity were also assessed (Fig. 3a and Additional file 1: Table S2), revealing that the highest rates of GBC were observed among American Indians and Alaska Natives in 2000–2018, while ICC, ECC and AVC incidence rates were highest among Asians and Pacific Islanders (using white patients as the reference, the incidence rate ratios for Black patients, American Indians and Alaskan Natives, and Asians and Pacific Islanders, were 1: 0.889, 1: 1.000, and 1: 1.333 for ICC, respectively; 1: 0.900, 1: 1.000, and 1: 1.400, respectively, for ECC; 1: 1.364, 1: 1.545, and 1: 1.182 for GBC, respectively; and 1: 0.833, 1: 0.667, and 1: 1.333, respectively, for AVC). From 2003 to 2018, ICC incidence rates rose among all analyzed ethnic groups other than the American Indian and Alaska Native population (White: APC, 8.1 [95% CI, 7.0–9.2]; Black: APC, 7.7 [95% CI, 6.2–9.2]; Asian and Pacific Islander: APC, 3.5 [95% CI, 2.1–4.9]).

BTC incidence rates were also assessed as a function of patient age (≥ 40 years) over the years (Fig. 3b), revealing significant increases in the incidence of all analyzed BTCs with age (ICC: AAPC, 7.2 [95% CI, 5.4–9.8]; ECC: AAPC, 9.0 [95% CI, 7.5–10.6]; GBC: AAPC, 8.3 [95% CI, 6.9–9.7]; AVC: AAPC, 7.5 [95% CI, 6.0–9.0]).

### Trends in BTC staging and treatment

**Fig. 4:**
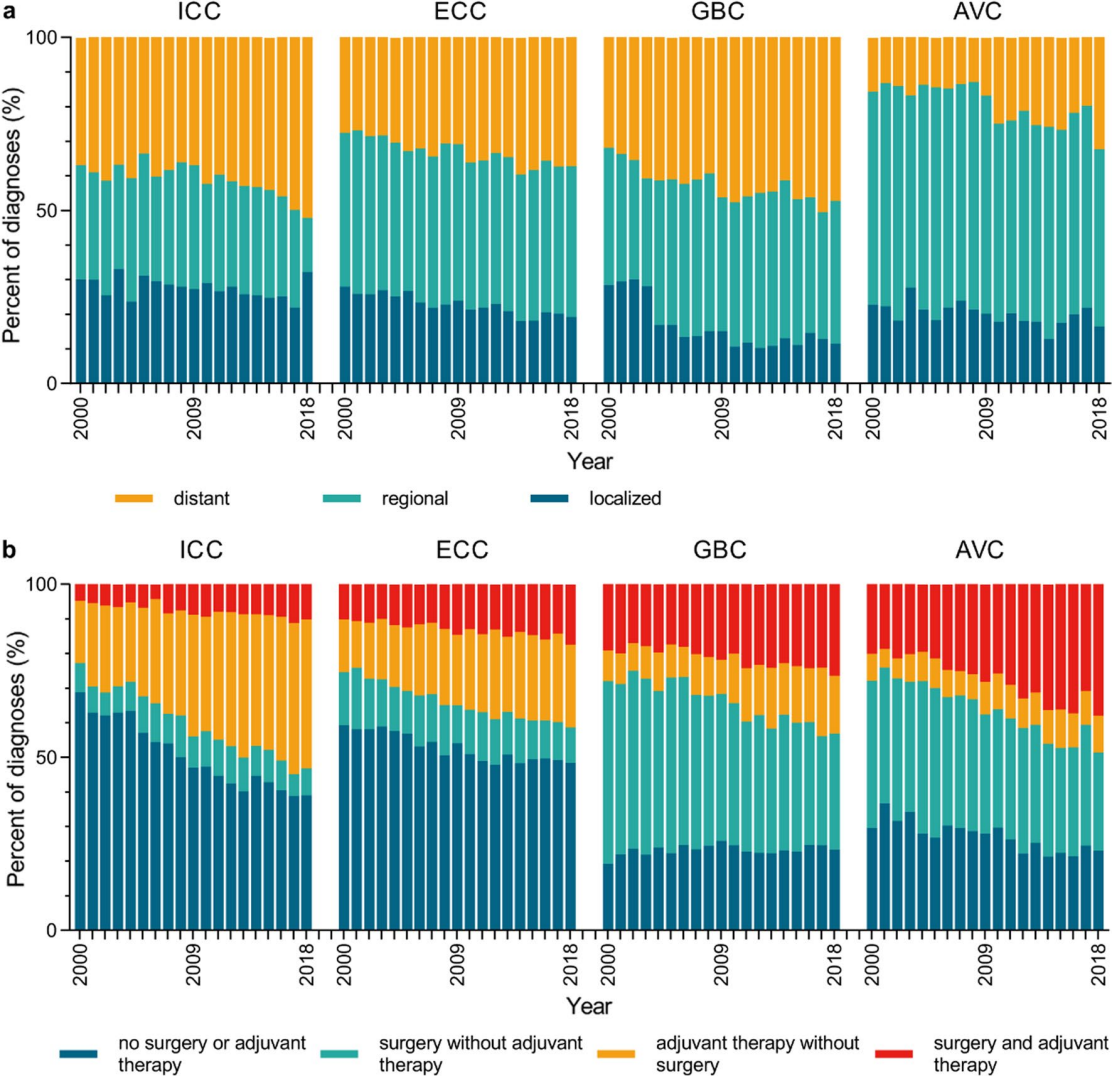
(**a**) Trends in BTC staging, 2000–2018. (**b**) Trends in BTC treatment, 2000–2018

The trends in BTC patient staging at initial presentation and treatment patterns are summarized in Fig. 4a and b, respectively. Overall, the percentage of BTC patients diagnosed with distant-stage disease rose with time (ICC: from 36.80% in 2000 to 52.10% in 2018; ECC: from 27.60% in 2000 to 37.20% in 2018;GBC: from 31.9% in 2000 to 47.2% in 2018; AVC: from 15.60% in 2000 to 32.30% in 2018), while the percentage of patients diagnosed with localized disease declined with time (ICC: from 30.10% in 2000 to 22.00% in 2017; ECC: from 27.60% in 2000 to 37.20% in 2018; GBC: from 28.4% in 2000 to 11.5% in 2018; AVC: from 22.70% in 2000 to 16.50% in 2018). For patients with AVC and GBC, surgery alone was the most common treatment strategy, while patients with ICC and ECC usually did not undergo either surgery nor adjuvant therapy. For patients with BTC, the percentage of patients that underwent surgery alone declined with time (ICC: from 8.40% in 2000 to 7.80% in 2018; ECC: from 15.40% in 2000 to 10.2% in 2018; GBC: from 52.70% in 2000 to 33.60% in 2018; AVC: from 42.50% in 2000 to 28.40% in 2018), while the percentage of patients that underwent adjuvant therapy alone or in combination with surgery rose with time (ICC: from 22.9% in 2000 to 53.2% in 2018; ECC: from 25.5% in 2000 to 41.3% in 2018; GBC: from 28.1% in 2000 to 43.1% in 2018; AVC: from 27.9% in 2000 to 48.6% in 2018).

### BTC patient survival trends and prognostic factors

**Fig. 5:**
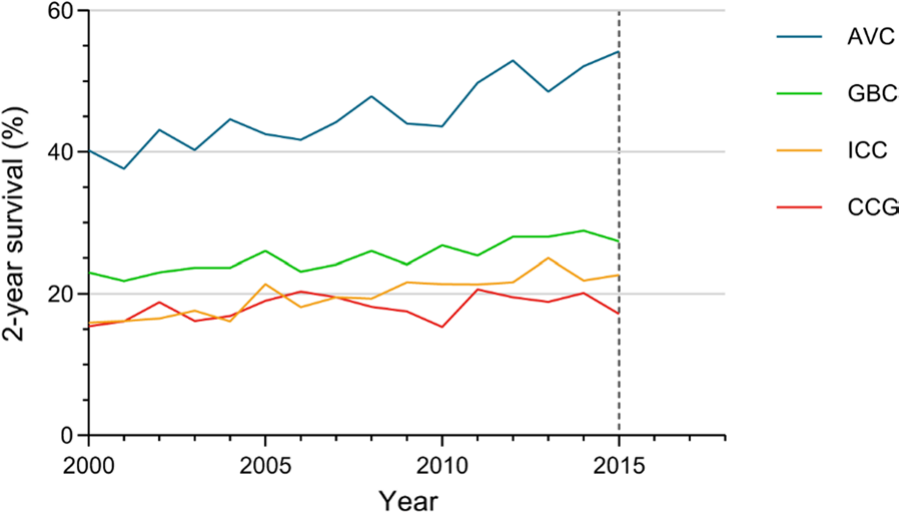
Trends in 2-year survival among BTC patients, 2000–2015

Lastly, the trends in the 2-year survival outcomes for BTC patients from 2000 to 2015 were assessed (Fig. 5). The survival rates for AVC and ECC patients were the highest and lowest, respectively, among the BTC patients, with corresponding average 2-year survival rates of 44.5 and 17.4%, respectively. The 2-year survival rates for ICC, GBC and AVC improved from 2000 to 2015 (ICC: from 15.90 to 22.70%; GBC: from 23.00 to 27.40%; AVC: from 40.20 to 54.20%). The ECC patient 2-year survival rates improved between 2000 and 2006 (from 15.4% in 2000 to 20.3% in 2006), and changed little between 2006 and 2014 (20.10% in 2014).

The variables of age, sex, race, marital status, grade, stage, and treatment pattern were incorporated into the Cox proportional hazards regression model (Additional file 1: Table S3). The results showed that age, grade, and treatment pattern were significant independent prognostic risk factors for BTC patients.

## Discussion

The present population-based study used data from the SEER registry to conduct the largest analysis of BTC patient data available through 2018. Overall, the BTC incidence continued to rise over the analyzed 19-year period, primarily due to increases in the rates of ICC, in line with the findings from previous epidemiological studies [13, 14]. We analyzed various aspects of this increased BTC incidence, finding that the trends differed according to the anatomical site of the tumor. As there were significant differences in survival for the different BTC types, direct comparisons with studies that have described overall BTC trends are likely to be inappropriate. The improvement of survival reflects the progress made in BTC management over the past few decades and, in the future, additional efforts should be made to improve the survival of BTC patients.

ICC is a heterogeneous disease, and its development has been associated with several risk factors including viral hepatitis, liver cirrhosis, and other systemic or gastrointestinal diseases [4, 21]. There are also marked geographical differences in the ICC incidence throughout the world, and epidemiological differences have been observed even when examining populations exposed to similar risk factors. These differences may be attributable to ICC misclassification in at least some cases. Consistently, ICCs have been diagnosed as cases of hepatocellular carcinoma, mixed cancer, or cancer of unknown primary origin, while Klatskin tumors are occasionally misclassified as ICC [4, 19, 22]. Definitive diagnosis of ICC in the more advanced stages of the disease is also challenging. Overall, ICC remains a relatively rare form of cancer and its misdiagnosis can lead to significant bias regarding the overall ICC incidence rates. Clearer diagnostic criteria are thus essential to guide the more accurate diagnosis of this disease. However, it is important to note that no identifiable risk factors are known to be associated with the observed increase in ICC incidence over time.

Over the 2000–2018 period, the rates of GBC, AVC, and ECC remained relatively stable in the USA, with some increases and decreases in incidence observed in certain years. Stable or decreasing ECC incidence has also been reported in Europe and Japan [23, 24]. One systematic review found that congenital cystic dilatations of the biliary tree were most strongly associated with ECC (OR 34.94, 95% CI, 24.36–50.12), although these dilatations are more common in females than males, in contrast to the observed ECC incidence pattern. Both choledocholithiasis and cholelithiasis are risk factors associated with ECC (OR 18.58, 95% CI, 11.07–31.18 and OR 5.92, 95% CI, 3.09–11.32; respectively), although there is insufficient available evidence to definitively establish whether alcohol intake, smoking, diabetes, overweight/obesity, or inflammatory bowel disease are also risk factors for this form of BTC [25].

Several known risk factors for GBC include gallstones, infections, gallbladder polyps, and other inflammatory conditions [8, 26]. The past study reported that due to Cholecystectomy population at risk of developing GBC decreased [27]. Given that the total cholecystectomy rates have remained largely unchanged since the mid-1990s, there were few changes in GBC incidence over the target study period [11]. The GBC incidence was substantially higher in females compared with males, and these gender-related differences observed in the USA are consistent with those observed in other regions of the world. One recent analysis found GBC susceptibility in females to be associated with variants in the prostate stem cell antigen gene [28].

Overall, AVC is a relatively rare form of cancer, accounting for just 15.1% of all BTC cases in the USA. One recent study found that the incidence of AVC incidence changed less among whites between 1992 and 2009, while declining among female Asians and Pacific Islanders [11]. In the present study, the AVC trends remained stable in all ethnic groups over the analyzed period (*P* ≥ 0.05). AVC-related risk factors have not been definitively established, although one recent study observed increased AVC incidence to be related to smoking but not alcohol intake [29]. This is consistent with the observed higher incidence of AVC in males, as males are more likely to be smokers.

Effective treatment of BTC remains a significant clinical challenge as patients often do not present for diagnosis until the disease is in an advanced stage [30]. Increases in the diagnosis of BTC with distant-stage disease rate of BTC diagnosis in receny years suggest that improvements in certain imaging-based diagnostic tools may have led to the detection of metastases that would previously have been overlooked. The rates of adjuvant therapy for BTC patients have also risen over the past 19 years, with several studies demonstrating a link between adjuvant therapy and improved BTC patient survival outcomes. For example, one systematic review and meta-analysis of 20 studies determined that adjuvant chemotherapy and adjuvant chemoradiation (OR, 0.39, 95% CI, 0.23- 0.66; 0.61, 95% CI, 0.38–0.99, respectively) yielded greater benefits in BTC patients who had undergone surgical tumor resection [31]. This is consistent with the 2-year survival rates observed in the present study, suggesting that increased use of adjuvant therapy is contributing to a better prognosis for individuals diagnosed with BTC. The survival rates were low among ECC patients as well as ICC patients, potentially due to the fact that these two forms of BTC are relatively difficult to resect. Overall, the mortality rates for patients with BTC have not declined substantially, suggesting that there has been little improvement in long-term patient prognosis. As the APC in ICC mortality was lower than that of the incidence, this suggests that the rise in patient 2-year survival may ultimately correspond to prolonged overall survival in this expanding patient population. Moreover, we performed multivariate survival analysis using the Cox proportional hazards regression model, finding that the independent prognostic factors of age, tumor grade, and treatment pattern affected the overall survival, which would be expected to facilitate clinical decision-making.

There are certain limitations to this analysis. First, the SEER database did not include all BTC cases. In addition, the diagnosis of BTC is often difficult and the classifications of other biliary tract tumors remains unclear. As a result, the observed incidence rate was almost certainly lower than the true incidence rate. In addition, data pertaining to patient vital exposure, diabetes status, smoking history, or history of biliary tract disease were not available, precluding any analyses of these risk factors. The proportion of BTC patients who did not receive treatment was not low, especially ICC and ECC, and the reasons for this lack of treatment are worthy of particular attention. Despite significant progress in the development of targeted therapies and immunotherapies in recent years, data from the SEER database could not be used to examine the relationship between these therapies and BTC patient survival outcomes [32, 33]. One recent study using the SEER database found that treatment with FDA-approved targeted therapies led to significant improvements in lung cancer patient 2-year survival since 2013 with corresponding reductions in mortality [34]. However, this was not observed in the present study.

## Conclusion

In summary, the results of this analysis of SEER registry data over a 19-year period revealed that both ICC incidence and mortality rates have risen with time, while the incidence and mortality of ECC, GBC, and AVC have remained relatively stable. These different forms of BTC exhibited significant differences in their epidemiology, staging at diagnosis, treatment, and prognosis. Both surgery and adjuvant therapy remain important approaches to the treatment of BTC patients, and the average 2-year survival rates for patients with these cancers have continued to improve with time. However, further research is required to determine the efficacy of targeted therapies and immunotherapeutic interventions in patients with BTC.

## Supplementary Information

**Additional file 1: Table S1.** Demographics and Clinical Characteristics of the Population. **Table S2.** Incidence rates (per 100000 person-year) by sex and race in 2000 to 2018. **Table S3.** Multivariate analyses for OS and CSS in CHC patients.

## Data Availability

All data are derived from the Surveillance, Epidemiology, and End Results (SEER) database produced by the National Cancer Institute (https://seer.cancer.gov/) Program.

## References

[1] Razumilava N, Gores GJ (2014). Cholangiocarcinoma. The Lancet.

[2] Razumilava N, Gores GJ (2011). Combination of gemcitabine and cisplatin for biliary tract cancer: a platform to build on. J Hepatol.

[3] Patel T (2002). Worldwide trends in mortality from biliary tract malignancies. BMC Cancer.

[4] Khan SA, Tavolari S, Brandi G (2019). Cholangiocarcinoma: epidemiology and risk factors. Liver Int.

[5] Fitzmaurice C, Dicker D, Pain A, Hamavid H, Moradi-Lakeh M, Global Burden of Disease Cancer C (2015). The global burden of cancer 2013. JAMA Oncol.

[6] Jan YY, Yeh CN, Yeh TS, Hwang TL, Chen MF (2005). Clinicopathological factors predicting long-term overall survival after hepatectomy for peripheral cholangiocarcinoma. World J Surg.

[7] Cho SY, Park SJ, Kim SH, Han SS, Kim YK, Lee KW (2010). Survival analysis of intrahepatic cholangiocarcinoma after resection. Ann Surg Oncol.

[8] Ertel AE, Bentrem D, Abbott DE (2016). Gall bladder cancer. Cancer Treat Res.

[9] Rizzo A, Frega G, Ricci AD, Palloni A, Abbati F, De Lorenzo S (2020). Anti-EGFR monoclonal antibodies in advanced biliary tract cancer: a systematic review and meta-analysis. In Vivo.

[10] Horgan AM, Knox JJ (2018). Adjuvant therapy for biliary tract cancers. J Oncol Pract.

[11] Castro FA, Koshiol J, Hsing AW, Devesa SS (2013). Biliary tract cancer incidence in the United States-Demographic and temporal variations by anatomic site. Int J Cancer.

[12] Torre LA, Siegel RL, Islami F, Bray F, Jemal A (2018). Worldwide burden of and trends in mortality from gallbladder and other biliary tract cancers. Clin Gastroenterol Hepatol.

[13] Valle JW, Kelley RK, Nervi B, Oh D-Y, Zhu AX (2021). Biliary tract cancer. The Lancet.

[14] Saha SK, Zhu AX, Fuchs CS, Brooks GA (2016). Forty-year trends in cholangiocarcinoma incidence in the U.S.: intrahepatic disease on the rise. Oncologist.

[15] Horgan AM, Amir E, Walter T, Knox JJ (2012). Adjuvant therapy in the treatment of biliary tract cancer: a systematic review and meta-analysis. J Clin Oncol.

[16] Primrose JN, Fox RP, Palmer DH, Malik HZ, Prasad R, Mirza D (2019). Capecitabine compared with observation in resected biliary tract cancer (BILCAP): a randomised, controlled, multicentre, phase 3 study. Lancet Oncol.

[17] Ghidini M, Tomasello G, Botticelli A, Barni S, Zabbialini G, Seghezzi S (2017). Adjuvant chemotherapy for resected biliary tract cancers: a systematic review and meta-analysis. HPB (Oxford).

[18] Rizzo A, Brandi G (2021). First-line chemotherapy in advanced biliary tract cancer ten years after the ABC-02 trial: "And Yet It Moves!". Cancer Treat Res Commun.

[19] Welzel TM, McGlynn KA, Hsing AW, O'Brien TR, Pfeiffer RM (2006). Impact of classification of hilar cholangiocarcinomas (Klatskin tumors) on the incidence of intra- and extrahepatic cholangiocarcinoma in the United States. J Natl Cancer Inst.

[20] Clegg LX, Hankey BF, Tiwari R, Feuer EJ, Edwards BK (2009). Estimating average annual per cent change in trend analysis. Stat Med.

[21] Tyson GL, El-Serag HB (2011). Risk factors for cholangiocarcinoma. Hepatology.

[22] Massarweh NN, El-Serag HB (2017). Epidemiology of hepatocellular carcinoma and intrahepatic cholangiocarcinoma. Cancer Control.

[23] Utada M, Ohno Y, Tamaki T, Sobue T, Endo G (2014). Long-term trends in incidence and mortality of intrahepatic and extrahepatic bile duct cancer in Japan. J Epidemiol.

[24] Bergquist A, von Seth E (2015). Epidemiology of cholangiocarcinoma. Best Pract Res Clin Gastroenterol.

[25] Clements O, Eliahoo J, Kim JU, Taylor-Robinson SD, Khan SA (2020). Risk factors for intrahepatic and extrahepatic cholangiocarcinoma: a systematic review and meta-analysis. J Hepatol.

[26] Nogueira L, Freedman ND, Engels EA, Warren JL, Castro F, Koshiol J (2014). Gallstones, cholecystectomy, and risk of digestive system cancers. Am J Epidemiol.

[27] Diehl AK, Beral V (1981). Cholecystectomy and changing mortality from gallbladder cancer. Lancet.

[28] Rai R, Sharma KL, Misra S, Kumar A, Mittal B (2013). PSCA gene variants (rs2294008 and rs2978974) confer increased susceptibility of gallbladder carcinoma in females. Gene.

[29] McGee EE, Jackson SS, Petrick JL, Van Dyke AL, Adami HO, Albanes D (2019). Smoking, alcohol, and biliary tract cancer risk: a pooling project of 26 prospective studies. J Natl Cancer Inst.

[30] Valle JW (2010). Advances in the treatment of metastatic or unresectable biliary tract cancer. Ann Oncol.

[31] Kish M, Chan K, Perry K, Ko YJ (2020). A systematic review and network meta-analysis of adjuvant therapy for curatively resected biliary tract cancers. Curr Oncol.

[32] Rizzo A, Ricci AD, Brandi G (2021). Recent advances of immunotherapy for biliary tract cancer. Expert Rev Gastroenterol Hepatol.

[33] Rizzo A, Ricci AD, Brandi G (2021). Futibatinib, an investigational agent for the treatment of intrahepatic cholangiocarcinoma: evidence to date and future perspectives. Expert Opin Investig Drugs.

[34] Howlader N, Forjaz G, Mooradian MJ, Meza R, Kong CY, Cronin KA (2020). The effect of advances in lung-cancer treatment on population mortality. N Engl J Med.

