# Supplementary material for: The epidemiological trends of biliary tract cancers in the United States of America
Source: BMC Gastroenterol. 2022 Dec 29;22:546. doi: 10.1186/s12876-022-02637-8 (PMC9801670; doi:10.1186/s12876-022-02637-8)
Supplement: Supplementary file 1 — Additional file 1: Table S1. Demographics and Clinical Characteristics of the Population. Table S2. Incidence rates (per 100000 person-year) by sex and race in 2000 to 2018. Table S3. Multivariate analyses for OS and CSS in CHC patients. [file 12876_2022_2637_MOESM1_ESM.docx]

**The epidemiological trends of biliary tract cancers in the United States of America**

**Yong Jiang^1^ · Liyong Jiang^1^ · Feiyu Li^1^ · Qingbin Li^2^ · Shuai Yuan^2^ · Songhan Huang ^1^ · Yingda Fu^1^ · Xiangyu Yan^1^ · Ji Chen^2^ · Hongxin Li^1^ · Shenhao Li^1^ · Jun liu^1, 2^**

^1^Department of Liver Transplantation and Hepatobiliary Surgery, Shandong Provincial Hospital, Shandong University, Jinan, China

^2^Department of Liver Transplantation and Hepatobiliary Surgery, Shandong Provincial Hospital Affiliated to Shandong First Medical University, Jinan, China

**Correspondence:**

Jun Liu

Add: No.324, Jingwu Road, Jinan, Shandong, China

Email: [dr_liujun1967@126.com](mailto:dr_liujun1967@126.com)

**Table S1.** Demographics and Clinical Characteristics of the Population

| **Characteristic** | **Total** (%) | **ICC** (%) | **ECC** (%) | **GBC** (%) | **AVC** (%) | **Other** (%) |
| --- | --- | --- | --- | --- | --- | --- |
| **n** | 64666 | 14830 | 17004 | 19187 | 9742 | 3903 |
| **Age, mean ± SD** | 70.03 ± 12.96 | 68.15 ± 13.04 | 71.33 ± 12.74 | 70.46 ± 12.80 | 69.29 ± 12.90 | 71.30 ± 13.37 |
| **Gender** |  |  |  |  |  |  |
| female | 34691 (53.6) | 7312 (49.3) | 9309 (54.7) | 10577 (55.1) | 5395 (55.4) | 2098 (53.8) |
| male | 29975 (46.4) | 7518 (50.7) | 7695 (45.3) | 8610 (44.9) | 4347 (44.6) | 1805 (46.2) |
| **Race** |  |  |  |  |  |  |
| White | 50589 (78.2) | 11720 (79) | 13334 (78.4) | 14689 (76.6) | 7703 (79.1) | 3143 (80.5) |
| Black | 6130 (9.5) | 1175 (7.9) | 1423 (8.4) | 2355 (12.3) | 819 (8.4) | 358 (9.2) |
| American Indian and Alaska Native | 625 (1) | 132 (0.9) | 147 (0.9) | 252 (1.3) | 61 (0.6) | 33 (0.8) |
| Asian and Pacific Islander | 7151 (11.1) | 1769 (11.9) | 2071 (12.2) | 1828 (9.5) | 1122 (11.5) | 361 (9.2) |
| unknown | 171 (0.3) | 34 (0.2) | 29 (0.2) | 63 (0.3) | 37 (0.4) | 8 (0.2) |
| **Marital status** |  |  |  |  |  |  |
| married (including common law) | 34021 (52,6) | 8231 (55.5) | 9171 (53.9) | 9197 (47.9) | 5536 (56.8) | 1886 (48.3) |
| divorced / separated | 6020 (9.3) | 1525 (10.3) | 1483 (8.7) | 1769 (9.2) | 871 (8.9) | 372 (9.5) |
| married (including common law) | 34021 (52,6) | 8231 (55.5) | 9171 (53.9) | 9197 (47.9) | 5536 (56.8) | 1886 (48.3) |
| single (never married) | 8347 (12.6) | 1991 (13.4) | 2057 (12.1) | 2553 (13.3) | 1209 (12.4) | 537 (13.8) |
| widowed / unknown | 16278 (25.2) | 3083 (20.8) | 4293 (25.2) | 5668 (29.5) | 2126 (21.8) | 1108 (28.4) |
| **Grade** |  |  |  |  |  |  |
| I | 4125 (6.4) | 506 (3.4) | 830 (4.9) | 1704 (8.9) | 1011 (10.4) | 74 (1.9) |
| II | 13558 (21) | 2142 (14.4) | 2577 (15.2) | 4940 (25.7) | 3630 (37.3) | 269 (6.9) |
| III | 11909 (18.4) | 2048 (13.8) | 2239 (13.2) | 4970 (25.9) | 2336 (24) | 316 (8.1) |
| IV | 674 (1.0) | 86 (0.6) | 106 (0.6) | 361 (1.9) | 106 (1.1) | 15 (0.4) |
| unknown | 34400 (53.2) | 10048 (67.8) | 11252 (66.2) | 7212 (37.6) | 2659 (27.3) | 3229 (82.7) |
| **Stage** |  |  |  |  |  |  |
| localized | 11299 (17.5) | 3395 (22.9) | 3085 (18.1) | 2881 (15) | 1764 (18.1) | 174 (4.5) |
| regional | 22993 (35.6) | 3711 (25) | 6035 (35.5) | 7487 (39) | 5350 (54.9) | 410 (10.5) |
| distant | 21970 (34) | 5437 (36.7) | 4636 (27.3) | 7831 (40.8) | 1770 (18.2) | 2296 (58.8) |
| unknown / un-staged | 8404 (13) | 2287 (15.4) | 3248 (19.1) | 988 (5.1) | 858 (8.8) | 1023 (26.2) |
| **Therapy** |  |  |  |  |  |  |
| no surgery or adjuvant therapy | 25514 (39.5) | 6935 (46.8) | 8915 (52.4) | 4473 (23.3) | 2638 (27.1) | 2553 (65.4) |
| surgery without adjuvant therapy | 15158 (23.4) | 1319 (8.9) | 2197 (12.9) | 8028 (41.8) | 3517 (36.1) | 97 (2.5) |
| adjuvant therapy without surgery | 13505 (20.9) | 5322 (35.9) | 3632 (21.4) | 2527 (13.2) | 851 (8.7) | 1173 (30.1) |
| both surgery and adjuvant therapy | 10489 (16.2) | 1254 (8.5) | 2260 (13.3) | 4159 (21.7) | 2736 (28.1) | 80 (2) |

Abbreviations: *ICC*, intrahepatic cholangiocarcinoma; *ECC*, extrahepatic cholangiocarcinoma; *GBC*, gallbladder cancer; *AVC*, ampulla of Vater cancer; *Other*, other biliary tract cancers; Grade *I*, well differentiated; Grade *II*, moderately differentiated; Grade *III*, poorly differentiated; Grade *IV* , undifferentiated.

**Table S2.** Incidence rates (per 100000 person-year) by sex and race in 2000 to 2018

| **Characteristic** | **ICC** | | **ECC** | | **GBC** | | **AVC** | |
| --- | --- | --- | --- | --- | --- | --- | --- | --- |
|  | **Incidence rate** | **ratio** | **Incidence rate** | **ratio** | **Incidence rate** | **ratio** | **Incidence rate** | **ratio** |
| **Gender** |  |  |  |  |  |  |  |  |
| male | **1.1** | reference | **1.2** | reference | 0.9 | reference | **0.7** | reference |
| female | 0.8 | 0.727 | 0.8 | 0.667 | **1.4** | 1.556 | 0.5 | 0.714 |
| **Race** |  |  |  |  |  |  |  |  |
| White | 0.9 | reference | 1.0 | reference | 1.1 | reference | 0.6 | reference |
| Black | 0.8 | 0.889 | 0.9 | 0.900 | 1.5 | 1.364 | 0.5 | 0.833 |
| American Indian and Alaska Native | 0.9 | 1.000 | 1.0 | 1.000 | **1.7** | 1.545 | 0.4 | 0.667 |
| Asian or Pacific Islander | **1.2** | 1.333 | **1.4** | 1.400 | 1.3 | 1.182 | **0.8** | 1.333 |

Bold incidence rates are considered higher or th highest rates.

Abbreviations: *ICC*, intrahepatic cholangiocarcinoma; *ECC*, extrahepatic cholangiocarcinoma; *GBC*, gallbladder cancer; *AVC*, ampulla of Vater cancer.

**Table S3.** Multivariate analyses for OS and CSS in CHC patients

| **Characteristic** | **ICC** | | **ECC** | | **GBC** | | **AVC** | |
| --- | --- | --- | --- | --- | --- | --- | --- | --- |
|  | **Hazard ratio (95% CI)** | ***P*** | **Hazard ratio (95% CI)** | ***P*** | **Hazard ratio (95% CI)** | ***P*** | **Hazard ratio (95% CI)** | ***P*** |
| **Age** |  |  |  |  |  |  |  |  |
| <70 | reference |  |  |  |  |  |  |  |
| ≥70 | 1.257 (1.208-1.308) | **<0.001** | 1.324 (1.276-1.373) | **<0.001** | 1.426 (1.377-1.478) | **<0.001** | 1.569 (1.485-1.657) | **<0.001** |
| **Gender** |  |  |  |  |  |  |  |  |
| female | reference |  |  |  |  |  |  |  |
| male | 1.154 (1.112-1.198) | **<0.001** | 0.983 (0.952-1.016) | 0.308 | 0.986 (0.955-1.018) | 0.390 | 1.029 (0.980-1.080) | 0.248 |
| **Race** |  |  |  |  |  |  |  |  |
| White | reference |  |  |  |  |  |  |  |
| Black | 1.022 (0.955-1.094) | 0.526 | 1.049 (0.989-1.112) | 0.113 | 1.019 (0.970-1.070) | 0.455 | 1.178 (1.080-1.285) | **<0.001** |
| American Indian and Alaska Native | 1.229 (1.021-1.479) | **0.029** | 1.025 (0.861-1.221) | 0.778 | 1.002 (0.873-1.149) | 0.982 | 1.092 (0.805-1.480) | 0.571 |
| Asian and Pacific Islander | 0.870 (0.823-0.920) | **<0.001** | 0.897 (0.853-0.944) | **<0.001** | 0.882 (0.835-0.933) | **<0.001** | 0.867 (0.801-0.939) | **<0.001** |
| unknown | 0.457 (0.275-0.758) | **0.002** | 0.350 (0.188-0.651) | **<0.001** | 0.498 (0.339-0.731) | **<0.001** | 0.297 (0.141-0.623) | **0.001** |
| **Marital status** |  |  |  |  |  |  |  |  |
| married (including common law) | reference |  |  |  |  |  |  |  |
| single (never married) | 1.055 (0.998-1.115) | 0.059 | 1.103 (1.047-1.163) | **<0.001** | 1.133 (1.078-1.191) | **<0.001** | 1.065 (0.986-1.152) | 0.110 |
| divorced / separated | 1.099 (1.033-1.169) | **0.003** | 1.134 (1.068-1.203) | **<0.001** | 1.066 (1.007-1.128) | **0.029** | 1.076 (0.986-1.174) | 0.098 |
| widowed / unknown | 1.043 (0.992-1.095) | 0.098 | 1.076 (1.033-1.120 | **<0.001** | 1.122 (1.080-1.166) | **<0.001** | 1.031 (0.970-1.096) | 0.329 |
| **Grade** |  |  |  |  |  |  |  |  |
| I | reference |  |  |  |  |  |  |  |
| II | 1.408 (1.259-1.575) | **<0.001** | 1.154 (1.057-1.259) | **0.001** | 1.360 (1.272-1.454) | **<0.001** | 1.245 (1.138-1.362) | **<0.001** |
| III | 1.828 (1.635-2.044) | **<0.001** | 1.440 (1.318-1.574) | **<0.001** | 1.973 (1.845-2.110) | **<0.001** | 1.729 (1.574-1.900) | **<0.001** |
| IV | 1.975 (1.545-2.525) | **<0.001** | 1.362 (1.101-1.684) | **0.004** | 2.188 (1.932-2.479) | **<0.001** | 1.442 (1.140-1.824) | **0.002** |
| unknown | 1.570 (1.414-1.743) | **<0.001** | 1.247 (1.148-1.355) | **<0.001** | 1.460 (1.359-1.568) | **<0.001** | 1.133 (1.030-1.246) | **0.011** |
| **Stage** |  |  |  |  |  |  |  |  |
| localized | reference |  |  |  |  |  |  |  |
| regional | 1.554 (1.471-1.641) | **<0.001** | 1.385 (1.317-1.457) | **<0.001** | 1.816 (1.718-1.918) | **<0.001** | 1.466 (1.365-1.575) | **<0.001** |
| distant | 2.104 (1.995-2.219) | **<0.001** | 2.185 (2.076-2.300) | **<0.001** | 3.921 (3.688-4.169) | **<0.001** | 2.327 (2.136-2.534) | **<0.001** |
| unknown/unstaged | 1.326 (1.242-1.415) | **<0.001** | 1.153 (1.093-1.216) | **<0.001** | 1.771 (1.609-1.949) | **<0.001** | 1.225 (1.113-1.349) | **<0.001** |
| **Therapy** |  |  |  |  |  |  |  |  |
| no surgery or adjuvant therapy | reference |  |  |  |  |  |  |  |
| surgery without adjuvant therapy | 0.224 (0.207-0.243) | **<0.001** | 0.317 (0.297-0.338) | **<0.001** | 0.390 (0.370-0.412) | **<0.001** | 0.220 (0.205-0.237) | **<0.001** |
| adjuvant therapy without surgery | 0.401 (0.384-0.419) | **<0.001** | 0.481 (0.461-0.503) | **<0.001** | 0.425 (0.403-0.449) | **<0.001** | 0.546 (0.499-0.597) | **<0.001** |
| both surgery and adjuvant therapy | 0.186 (0.171-0.202) | **<0.001** | 0.247 (0.231-0.265) | **<0.001** | 0.265 (0.250-0.281) | **<0.001** | 0.197 (0.182-0.214) | **<0.001** |

Bold *P* values are considered statistically significant

Abbreviations: *ICC*, intrahepatic cholangiocarcinoma; *ECC*, extrahepatic cholangiocarcinoma; *GBC*, gallbladder cancer; *AVC*, ampulla of Vater cancer; Grade *I*, well differentiated; Grade *II*, moderately differentiated; Grade *III*, poorly differentiated; Grade *IV* , undifferentiated.
